# New faunistic records of the family Mycetophilidae (Insecta, Diptera) from Morocco

**DOI:** 10.3897/zookeys.934.49157

**Published:** 2020-05-19

**Authors:** Ouarda Banamar, Peter J. Chandler, Ouafaa Driauach, Boutaïna Belqat

**Affiliations:** 1 Department of Biology, Faculty of Sciences, University Abdelmalek Essaâdi, BP 2121, Tétouan, Morocco University Abdelmalek Essaâdi Tétouan Morocco; 2 606B Berryfield Lane, Melksham, Wilts SN12 6EL, UK Unaffiliated Melksham United Kingdom

**Keywords:** Beni Snassen, checklist, fungus gnats, Middle Atlas, Mycetophilidae, North Africa, Rif

## Abstract

A total of 54 species of Mycetophilidae are recorded for the first time in Morocco, of which 38 species are new to North Africa. A first checklist of Moroccan Mycetophilidae is appended, containing 64 species in 25 genera.

## Introduction

The family Mycetophilidae belongs to the superfamily Sciaroidea and is the most abundant and diverse family of fungus gnats worldwide, comprising more than 4500 species (Pape et al. 2011). Adult fungus gnats are associated with humid areas, especially moist woodlands. They are usually found in shady and moist habitats such as cavities and root systems of fallen trees, overhanging stream banks, and among undergrowth of woods (Søli et al. 2000). As larvae, most mycetophilids develop in fungal fruiting bodies, both terrestrial and saproxylic, or in fungal mycelia in dead wood and soil litter, while a few species develop in myxomycetes, and others in rotten wood, bryophytes, bird’s nests, or caves (Hutson et al. 1980; Yakovlev 1994).

The fungus gnat fauna of Morocco is practically unknown. The first information on Moroccan Mycetophilidae dates back to Séguy (1941) who described a new species *Rymosia exornata* based on a female collected in the High Atlas and which was placed later in synonymy with *Exechia fulva* Santos Abreu, 1920 by Chandler and Ribeiro (1995).

Five species, without given localities, were recorded by Chandler (1994): *Leia bimaculata* (Meigen, 1804), *Mycetophila sordida* van der Wulp, 1874, *Phronia biarcuata* (Becker, 1908), *Rymosia beaucournui* Matile, 1963, and *Rymosia pseudocretensis* Burghele-Balacesco, 1966. The localities on which these records were based are given here.

Three other species were reported by Chandler and Ribeiro (1995) without specifying localities: *Cordyla crassicornis* Meigen, 1818, *Mycetophila britannica* Laštovka & Kidd, 1975, and *Mycetophila pictula* Meigen, 1830. The localities on which the records of *C. crassicornis* and *M. pictula* were based are also given here.

Recent records were added by Chandler and Blasco-Zumeta (2001) who described *Sciophila iberolutea* Chandler & Blasco-Zumeta, 2001 from Spain and also recorded specimens of the species in Morocco (Oued y Kern, locality). Thus, prior to the present study, there were 10 species recorded from Morocco.

This paper is the first contribution relating specifically to the Mycetophilidae species of Morocco. New findings increase the number of Moroccan Mycetophilidae to 64. Of these, 54 species of Mycetophilidae are recorded for the first time in Morocco of which 38 species are new to North Africa.

## Material and methods

A total of 724 specimens of Mycetophilidae (576 males and 148 females) were collected using Malaise traps and by sweeping, between 2012 and 2018. Forty sites distributed over mountainous areas, such as the Rif, Beni Snassen, and Middle Atlas, were sampled.

Most of the material was collected by O. Banamar, O. Driauach, and B. Belqat. Additional material was provided by Dr M. Ebejer that he had collected in Morocco and some Moroccan Mycetophilidae deposited in the Natural History Museum London, UK (**BMNH**) and the Musée National d’Histoire Naturelle, Paris, France (**MNHN**). All the material preserved in 70% ethanol, was identified by P. Chandler. If not otherwise stated, the examined material is deposited in the Abdelmalek Essaâdi University, Tétouan, Diptera collection.

**Figure 1.**
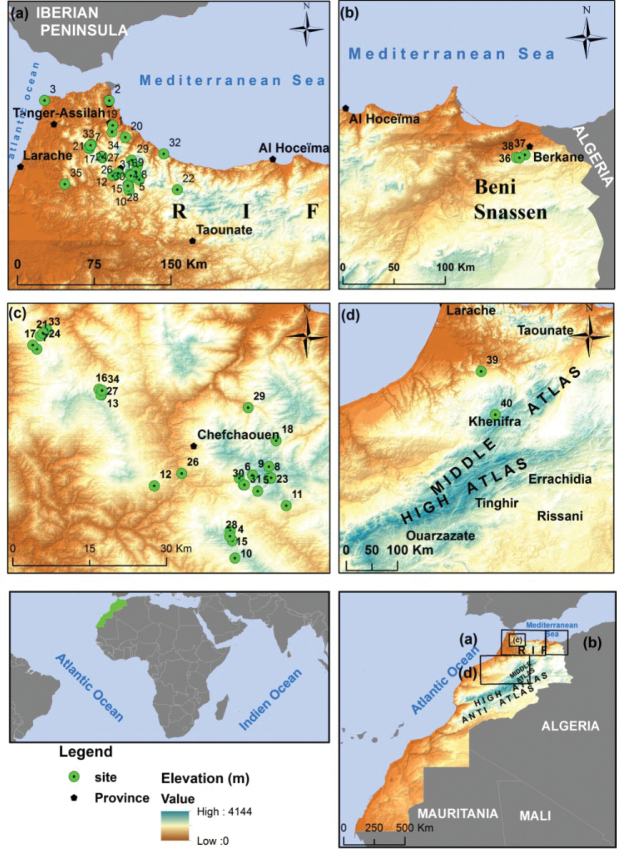
Map showing all collecting localities for Mycetophilidae in this study; numbers correspond to those in the Table 1.

A list of sampling sites, with coordinates and altitudes, is given in Table 1, and the locations of the sites are shown in Figure 1, which was prepared with ArcGis (version 9.3).

A first checklist of Moroccan Mycetophilidae, in alphabetical order, is given to summarize the species inventory presently known from Morocco. One (*) asterisk indicates that the species is the first record from Morocco; two (**) asterisks indicate that the species represents the first record for Morocco and North Africa.

**Table 1.** Sampling sites (in alphabetical order) harbouring the species collected in Morocco, in the present study, with localities, geographical coordinates and altitudes.

Province	Station	Locality	Elevation (m)	Geographical coordinates
**Rif**				
Tétouan	Marabout El Khaloua	Dar Khennouss	788	35°29.039'N, 5°20.678'W
Tanger	Grotte d’Hercule	Tanger	12	35°45.566'N, 5°56.350'W
Chefchaouen	Aïn El Ma Bared	Bouzthate	1267	35°00.333'N, 5°12.105'W
	Aïn El Malâab	Parc National Talassemtane	1278	35°05.509'N, 5°09.443'W
	Aïn Ras El Ma	Majjou	856	35°06.873'N, 5°11.388'W
	Aïn Sidi Brahim Ben Arrif	Larache	897	35°20.398'N, 5°32.712'W
	Aïn Takhninjoute	Bab Rouida	1512	35°06.881'N, 5°08.270'W
	Aïn Tiouila	Parc National Talassemtane	1502	35°07.194'N, 5°09.978'W
	Bab El Karne	Tamakoute	1248	34°58.510'N, 5°11.838'W
	Cascade Chrafate	Chrafate	820	35°03.997'N, 5°06.434'W
	Daya Afersiw	Mezine	303	35°06.069'N, 5°20.337'W
	Daya Amsemlil	Jbel Bouhachem	1059	35°15.596'N, 5°25.917'W
	Daya avant Taïda	Taïda	425	35°22.426'N, 5°31.662'W
	Daya Fifi	Fifi	1202	35°01.367'N, 5°12.335'W
	Daya Jbel Zemzem	Jbel Zemzem	216	35°45.457'N, 5°22.189'W
	Daya Mtahen	Jbel Bouhachem	966	35°16.195'N, 5°26.158'W
	Daya Tazia	Route Moulay Abdessalam	721	35°20.814'N, 5°33.139'W
	Douar Abou Boubnar (Marabout Sidi Gile)	Parc National Talassemtane	1247	35°10.812'N, 5°07.500'W
	Douar Kitane	Kitane	52	35°32.412'N, 5°20.393'W
	Douar Tizga	Amsa	516	35°26.237'N, 5°13.694'W
	Forêt-Adrou	Parc National Bouhachem	580	35°13.538'N, 5°19.405'W
	Forêt-Aïn Boughaba	Jbel Bou Bessoui	1526	34°58.779'N, 4°46.366'W
	Forêt-Jbel Lekraa	Parc National Talassemtane	1541	35°06.825'N, 5°08.077'W
	Forêt-Taghzoute	Parc National Bouhachem	556	35°22.056'N, 5°32.034'W
	Maison forestière	Parc National Talassemtane	1674	35°08.076'N, 5°08.262'W
	Oued Aârate	Dardara	269	35°07.381'N, 5°17.456'W
	Oued Amsemlil	Jbel Bouhachem	1059	35°15.614'N, 5°25.943'W
	Oued à 15 Km de Fifi	Bouzthate	1256	35°00.805'N, 5°12.365'W
	Oued Kelâa	Akchour	303	35°14.268'N, 5°10.452'W
	Oued Majjou	Majjou Village	799	35°06.186'N, 5°10.935'W
	Oued Majjou (Hafa Meqlouba)	Chefchaouen	825	35°06.175'N, 5°10.836'W
	Oued Sidi Yahya Aârab	Sidi Yahya Aârab	62	35°17.545'N, 4°53.503'W
	Oued Tisgris	Hmmadesh	490	35°22.079'N, 5°32.064'W
	Oued Tkarâa	Jbel Bouhachem	959	35°16.063'N, 5°25.829'W
	Route Ksar El Kébir-Chefchaouen	Tattofte	133	35°01.735'N, 5°45.593'W
**Beni Snassen (Eastern Morocco)**				
Berkane	Grotte des Pigeons	Beni Snassen	676	34°49.044'N, 5°24.329'W
	Grotte du Chameau	Beni Snassen	427	34°50.447'N, 2°21.532'W
	Oued Tafoughalt	Tafoughalt	751	34°48.941'N, 2°24.471'W
**Middle Atlas**				
Meknès	Aïn Walili	Sidi Moulay Idriss Zerhoun	490	34°03.216'N, 5°31.050'W
Ifrane	Forêt-3.5 km S. Azrou	Azrou	1450	33°25.491'N, 5°12.393'W

## List of species

### Family Mycetophilidae

#### Subfamily Mycetophilinae

**Tribe Exechiini**

**Genus *Allodiopsis* Tuomikoski, 1966**

##### * Allodiopsis rustica

Taxon classificationAnimaliaDipteraMycetophilidae

Edwards, 1941

F9F9C9E5-F022-5373-9185-9BE28F2C1038

###### Literature records.

Cited from North Africa (Tunisia) by Chandler et al. (2005).

###### New record.

Rif: Daya Tazia, 1♂ 12/V/2015.

##### Genus *Anatella* Winnertz, 1864

###### ** Anatella concava

Taxon classificationAnimaliaDipteraMycetophilidae

Plassmann, 1990

E25BEE6B-341E-5A13-82D7-0A1DA7880637

####### New record.

Rif: Oued Aârate, 1♂, 26/III/2014.

##### Genus *Brevicornu* Marshall, 1896

###### * Brevicornu intermedium

Taxon classificationAnimaliaDipteraMycetophilidae

(Santos Abréu, 1920)

8CA05B50-CEDE-5A92-8E2D-5F4BB70CD22B

####### Literature records.

Cited from North Africa by Chandler and Ribeiro (1995).

####### New records.

Rif: Forêt-Jbel Lekraa, 1♂, 12/VI/2013, coll. Ebejer; Oued Tisgris, 1♂, 25/III/2014; Oued Aârate, 1♂, 26/III/2014; Daya Tazia, 1♂, 25/IV/2014; Oued Majjou (Majjou Village), 2♂♂, 10/V/2014; Douar Tizga, 1♂, 25/VI/2014; Maison forestière, 2♂♂, 5♀♀, 07/VI-17/VI/2014, Malaise trap; Daya Jbel Zemzem, 1♂, 2/II/2015; Aïn Takhninjoute, 1♂, 21/IV/2015; Forêt-Adrou, 1♂, 25/IV/2015, coll. Ebejer; Daya Amsemlil, 2♂♂, 23/IV/2016.

###### ** Brevicornu griseicolle

Taxon classificationAnimaliaDipteraMycetophilidae

Staeger, 1840

ED13DFCE-944F-5176-AC19-3631B9690A1C

####### New records.

Rif: Daya Fifi, 1♂, 23/XI/2012; Oued Aârate, 1♂, 26/III/2014, 2♂♂, 07/V/2015; Daya Tazia, 1♂, 25/IV/2014; Oued Majjou (Majjou Village), 3♂♂, 7♀♀, 10/V/2014; Aïn El Malâab, 2♂♂, 2♀♀, 17/V/2014; Aïn El Malâab, 12♂♂, 17/V/2014; Maison forestière, 1♂7♀♀, 18/V/2014, 6♂♂, 17/VI/2014; Douar Tizga, 1♂, 25/VI/2014; Aïn Takhninjoute, 1♂, 21/IV/2015; Daya Amsemlil 1♂, 2♀♀, 23/IV/2016; Daya Mtahen, 1♂, 16/V/2016; Daya avant Taïda, 1♂, 20/IV/2018.

###### * Brevicornu sericoma

Taxon classificationAnimaliaDipteraMycetophilidae

(Meigen, 1830)

814767B1-8C37-55DD-8C7D-5368967DB861

####### Literature records.

Recorded from North Africa (Tunisia) (Chandler and Ribeiro 1995; Chandler et al. 2005).

####### New records.

Rif: Chefchaouen, VII/1975, 1♂, coll. J. Beaucournu (MNHN); Daya Fifi, 1♂, 23/XI/2012; Forêt-Jbel Lekraa, 1♂, 12/VI/2013, coll. Ebejer; Daya Fifi, 2♂♂, 26/III/2014; Oued Majjou (Majjou Village), 1♂, 10/V/2014; Maison forestière, 17/VI/2014; Douar Tizga, 1♂, 25/VI/2014; Oued Amsemlil, 2♂♂, 28/II/2015; Grotte d’Hercule, 3♂♂, 29/III/2015; Forêt-Adrou, 1♂, 25/IV/2015, coll. Ebejer; Cascade Chrafate, 1♂, 28/IV/2015; Oued Aârate, 10♂♂, 07/V/2015; Bab El Karne, 2♂♂, 25/XII/2015; Daya Amsemlil, 2♂♂, 16/V/2016.

###### * Brevicornu verralli

Taxon classificationAnimaliaDipteraMycetophilidae

(Edwards, 1925)

FB80F98F-CCEF-5862-B5FF-EBC743EA9665

####### Literature records.

Recorded from North Africa (Tunisia) (Chandler 1994, Chandler and Ribeiro 1995).

####### New records.

Rif: Daya Afersiw, 1♂, 11/VI/2013; Forêt-Jbel Lekraa, 1♂, 12/VI/2013, coll. Ebejer.

##### Genus *Cordyla* Meigen, 1803

###### Cordyla crassicornis

Taxon classificationAnimaliaDipteraMycetophilidae

Meigen, 1818

ED844F24-A7BD-5BD4-9940-5B879AE67169

####### Literature records.

Recorded from Morocco (Chandler and Ribeiro 1995), based on 1 male in MNHN, which was from Chefchaouen, VII/1975, coll. J. Beaucournu.

####### New records.

Rif: Douar Abou Boubnar, 1♂, 18/V/2014; Maison forestière, 6♂♂, 1♀, 17/VI/2014, Forêt-Adrou, 1♂, 25/IV/2015, coll. Ebejer; Oued Sidi Yahya Aârab, 1♂, 1♀, 25/IV/2015, coll. Ebejer. Beni Snassen: Oued Tafoughalt, 1♂, 25/XII/2014.

###### ** Cordyla insons

Taxon classificationAnimaliaDipteraMycetophilidae

Laštovka & Matile, 1974

9AA64531-3D26-55CE-9E16-D4F8FA6BE558

####### New record.

Rif: Oued Majjou (Majjou Village), 1♂, 09/IV/2013.

###### ** Cordyla murina

Taxon classificationAnimaliaDipteraMycetophilidae

Winnertz, 1864

ADB9F17F-D869-52E7-9E57-BDDF50F56604

####### New record.

Rif: Forêt-Aïn Boughaba, 1♂, 24/V/2013.

###### ** Cordyla styliforceps

Taxon classificationAnimaliaDipteraMycetophilidae

(Bukowski, 1934)

2A6A875D-A0F6-5835-840D-372F0D07B45A

####### New records.

Rif: Maison forestière, 1♂, 4/VII/2013, 12♂♂, 17/VI/2014, 1♂, 17/III/2015; Oued Tkarâa, 1♂, 1♀, 16/V/2016; Oued Sidi Yahya Aârab, 1♂, 25/IV/2015.

##### Genus *Exechia* Winnertz, 1864

###### ** Exechia bicincta

Taxon classificationAnimaliaDipteraMycetophilidae

(Staeger, 1840)

33801F74-294D-5CBA-8EDE-C1074A9085F9

####### New records.

Rif: Oued Kelâa, 1♀, 13/II/2013; Oued Aârate, 1♀, 26/III/2014. Beni Snassen: Grotte des pigeons, 1♂, 25/XII/2014; Oued Tafoughalt, 1♂, 25/XII/2014.

###### ** Exechia dorsalis

Taxon classificationAnimaliaDipteraMycetophilidae

(Staeger, 1840)

F3BA71ED-54E3-543E-A31C-D1D2918D0E70

####### New record.

Rif: Bab El Karne, 1♂, 25/XII/2016.

###### Exechia fulva

Taxon classificationAnimaliaDipteraMycetophilidae

Santos Abreu, 1920

82E88997-2DE8-5C8D-AB92-489E5095E6EC

= Rymosia exornata Séguy in Séguy 1941: 26

####### Literature records.

Cited from Morocco: High Atlas, Toubkal (Séguy 1941, Chandler and Ribeiro 1995).

####### New records.

Rif: 20 km west of Targuist, Ketama, 23/IV/1966, 6♂♂, 3♀♀, coll. A.M. Hutson (BMNH); Chefchaouen, VII/1975, 21♂♂, 20♀♀, coll. J. Beaucournu (MNHN); Oued Kelâa, 4♂♂, 4♀♀, 13/II/2013; Forêt-Aïn Boughaba, 7♂♂, 5♀♀, 24/V/2013; Forêt-Jbel Lekraa, 1♂, 12/VI/2013, coll. Ebejer; Daya Fifi, 14♂♂, 11♀♀, 26/III/2014; Aïn Sidi Brahim Ben Arrif, 1♂, 25/IV/2014; Oued Majjou (Majjou Village), 1♂, 10/V/2014; Aïn Takhninjoute, 4♂♂, 1♀, 17/V/2014; Maison forestière, 37♂♂, 32♀♀, 07/VI-17/VI/2014, Malaise trap; Oued Amsemlil, 2♂♂, 3♀♀, 28/II/2015; Daya Jbel Zemzem, 1♂, 02/III/2015; Aïn Takhninjoute, 4♂♂, 21/IV/2015; Bab El Karne, 22♂♂, 4♀♀, 25/XII/2015; Daya Fifi, 3♂♂, 5♀♀, 25/XII/2015; Daya Amsemlil, 2♂♂, 23/IV/2016.

###### * Exechia fusca

Taxon classificationAnimaliaDipteraMycetophilidae

(Meigen, 1804)

70D3073A-1BB5-5553-A119-BC85A30EBD78

####### Literature records.

Cited from North Africa (Tunisia) (Chandler 1994; Chandler and Ribeiro 1995).

####### New records.

Rif: Oued Kelâa, 2♂♂, 13/II/2013; Forêt-Jbel Lekraa, 1♂, 12/VI/2013, coll. Ebejer; Douar Kitane, 1♂, 01/I/2015; Daya Amsemlil, 1♂, 23/IV/2016.

##### Genus *Exechiopsis* Tuomikoski, 1966

###### * Exechiopsis coremura

Taxon classificationAnimaliaDipteraMycetophilidae

(Edwards, 1928)

A8674904-4AA2-5D91-BD88-FB156716084A

####### Literature records.

Cited from North Africa (Algeria) (Hackman et al. 1988).

####### New record.

Rif: Cascade Chrafate, 1♂, 1♀, 28/IV/2015.

##### Genus *Pseudexechia* Tuomikoski, 1966

###### ** Pseudexechia tuomikoskii

Taxon classificationAnimaliaDipteraMycetophilidae

(Kjærandsen, 2009)

2760C1C7-EA5A-5148-85E0-2CFE17E9EEE9

####### New record.

Rif: Source Aheramen, 1♂, 10/V/2014.

##### Genus *Rymosia* Winnertz, 1864

###### * Rymosia affinis

Taxon classificationAnimaliaDipteraMycetophilidae

Winnertz, 1864

45A54B78-1460-5CD5-859F-830B3022533B

####### Literature records.

Cited from North Africa (Algeria) (Burghele-Balacesco 1967).

####### New records.

Rif: Maison forestière, 1♀, 17/VI/2014; Daya Amsemlil, 1♂, 28/II/2015; Aïn Tiouila, 1♂, 02/V/2015; Daya Fifi, 1♂, 25/XII/2015.

###### Rymosia beaucournui

Taxon classificationAnimaliaDipteraMycetophilidae

Matile, 1963

DB8DCB22-5F02-55F2-ADA9-5AC98D261F55

####### Literature records.

Cited from Morocco (Chandler and Ribeiro 1995; Chandler et al. 2005). Records of the species (9 males, 3 females) based on specimens in MNHN from Rabat, Oued y Kern, V/1973, coll. H. Choumara.

####### New record.

Beni Snassen: Grotte des pigeons, 1♂, 25/XI/2014.

###### Rymosia pseudocretensis

Taxon classificationAnimaliaDipteraMycetophilidae

Burghele-Balacesco, 1966

DDAC34A5-7D2D-52E9-B51C-D64080150BF8

####### Literature records.

Cited from Morocco (Chandler 1994; Chandler et al. 2005), based on a specimen (1 male) in MNHN from Rabat, Oued y Kern, V/31973, coll. H. Choumara.

##### Genus *Stigmatomeria* Tuomikoski, 1966

###### ** Stigmatomeria crassicornis

Taxon classificationAnimaliaDipteraMycetophilidae

(Stannius, 1831)

BFC4DFA4-F966-599D-84A0-8D0C2EA50BDD

####### New records.

Rif: Chefchaouen, VII/1975, 1♀, coll. J. Beaucournu, MNHN; Forêt-Jbel Lekraa, 1♂, 12/VI/2013, coll. Ebejer; Daya Fifi, 1♀, 26/III/2014; Aïn Takhninjoute, 1♂, 17/V/2014; Maison forestière, 1♂, 18/V/2014, 1♀, 17/VI/2014; Daya Amsemlil, 1♂, 23/IV/2016.

##### Genus *Tarnania* Tuomikoski, 1966

###### * Tarnania dziedzickii

Taxon classificationAnimaliaDipteraMycetophilidae

(Edwards, 1941)

25EF3E8A-B90C-522A-B3A8-B24B2C4E73F5

####### Literature records.

Cited from North Africa (Algeria) (Burghele-Balacesco 1967).

####### New records.

Rif: Maison forestière, 1♀, 18/V/2014; Daya Amsemlil, 1♂, 28/II/2015; Cascade Chrafate, 1♂, 1♀, 28/IV/2015; Daya Amsemlil, 1♀, 23/IV/2016. Grotte-Aïn El-Aouda, 1♂, 1♀, 2/V/1914, MNHN.

##### Tribe Mycetophilini

###### ** Mycetophila alea

Taxon classificationAnimaliaDipteraMycetophilidae

Laffoon, 1965

E638D47B-2230-5537-A57B-F750817415B3

####### New records.

Rif: Chefchaouen, VII/1975, 13♂♂, 6♀♀, coll. J. Beaucournu, MNHN; Aïn El Ma Bared, 1♀, 06/V/2014; Maison forestière, 1♂, 3♀♀, 17/VI/2014; Oued Aârate, 3♂♂, 07/V/2015; Bab El Karne, 2♂♂, 25/XII/2015.

###### Mycetophila britannica

Taxon classificationAnimaliaDipteraMycetophilidae

Laštovka & Kidd, 1975

BBC6EBCE-CF23-56F9-AD7B-02CF716B8C36

####### Literature records.

Recorded from Morocco (Chandler and Ribeiro 1995), based on material in MNHN, for which precise locality data was not noted.

####### New records.

Rif: Forêt-Aïn Boughaba, 2♂♂, 24/V/2013; Forêt-Jbel Lekraa, 2♂♂, 1♀, 12/VI/2013, coll. Ebejer; Douar Kitane, 1♂, 13/III/2014, Malaise trap; Aïn El Malâab, 11♂♂, 17-V-2014; Aïn Takhninjoute, 1♂, 17/V/2014; Oued à 15 km de Fifi, 2♂♂, 06/V/2014; Aïn El Ma Bared, 3♀♀, 06/V/2014; Maison forestière, 2♀♀, 18/V/2014; Oued Majjou (Hafa Meqlouba), 1♂, 10/V/2014; Oued Majjou (Majjou Village), 1♂, 10/V/2014; Aïn Takhninjoute, 1♂, 17/V/2014; Maison forestière, 2♀♀, 17/VI/2014; Grotte d’Hercule, 3♂♂, 2♀♀, 29/III/2015; Aïn Takhninjoute, 1♂, 21/IV/2015, 1♂, 24/VI/2015; Oued Aârate, 1♂, 07/V/2015; Bab El Karne, 1♂, 25/XII/2015; Daya Amsemlil, 1♀, 1♂, 23/IV/2016; Daya Mtahen, 1♂, 16/V/2016; Daya avant Taïda, 1♀, 20/IV/2018; Forêt-Taghzoute, 1♂1♀, 25/IV/2015, coll. Ebejer.

###### ** Mycetophila deflexa

Taxon classificationAnimaliaDipteraMycetophilidae

Chandler, 2001

F06256FE-1670-5749-81F5-7E966EEDCE0F

####### New records.

Forêt-Taghzoute, 1♂, 25/IV/2015; Route Ksar El Kébir-Chefchaouen, 2♂♂, 5/VI/2013, coll. Ebejer.

###### ** Mycetophila edwardsi

Taxon classificationAnimaliaDipteraMycetophilidae

Lundström, 1913

A5DD2191-BA31-5AFC-953C-722F1A313164

####### New records.

Rif: Daya Fifi, 2♂♂, 26/III/2014; Daya Tazia, 1♀, 25/IV/2014; Oued Tkarâa, 1♀, 16/V/2016; Forêt-Jbel Lekraa, 1♀, 12/VI/2013, coll. Ebejer; Forêt-Taghzoute, 1♀, 25/IV/2015, coll. Ebejer. Beni Snassen: Grotte d’Hercule, 1♀, 29/III/2015.

###### ** Mycetophila formosa

Taxon classificationAnimaliaDipteraMycetophilidae

Lundström, 1911

75BBAEA6-174B-56CF-BC37-19D26A62A591

####### New records.

Rif: Forêt-Jbel Lekraa, 1♂, 12/VI/2013, coll. Ebejer; Oued Amsemlil, 1♂, 28/II/2015, Daya Amsemlil, 1♂, 25/XII/2015; Daya Amsemlil, 2♂♂, 23/IV/2016; Daya avant Taïda, 1♂, 20/IV/2018.

###### ** Mycetophila marginata

Taxon classificationAnimaliaDipteraMycetophilidae

Winnertz, 1864

46F8D78F-B71F-55B6-A800-70A20E650376

####### New records.

Rif: Chefchaouen, VII/1975, 4♂♂, coll. J. Beaucournu, MNHN; Aïn Ras El Ma, 1♂, 3/V/2013; Oued Majjou (Majjou Village), 3♂♂, 10/V/2014; Maison forestière, 1♂, 17/VI/2014; Daya Amsemlil, 3♂♂, 23/IV/2016.

###### * Mycetophila perpallida

Taxon classificationAnimaliaDipteraMycetophilidae

Chandler, 1993

3B64392C-E72C-52AA-B3BE-B7FC46843E08

####### Literature records.

Cited from North Africa (Chandler 2009).

####### New records.

Rif: Forêt-Aïn Boughaba, 1♂, 1♀, 24/V/2013; Aïn El Malâab, 1♂, 14/VII/2013; Maison forestière, 2♂♂, 17/VI/2014; Bab El Karne, 1♂, 25/XII/2015; Daya Amsemlil, 3♂♂, 1♀, 23/IV/2016; Douar Kitane, 5♂♂, 1♀, 29/XII/2016.

###### Mycetophila pictula

Taxon classificationAnimaliaDipteraMycetophilidae

Meigen, 1830

B28D32E7-4716-5A03-A8C4-7F0FD34864DB

####### Literature record.

Cited from Morocco (Chandler and Ribeiro 1995), based on 1 male and 2 females in MNHN, which were from Chefchaouen, VII/1975, coll. J. Beaucournu.

####### New record.

Rif: Forêt-Jbel Lekraa, 1♀, 12/VI/2013, coll. Ebejer; Oued Majjou (Majjou Village), 1♂, 10/V/2014.

###### Mycetophila sordida

Taxon classificationAnimaliaDipteraMycetophilidae

van der Wulp, 1874

A6B7C65E-E4E1-5C64-8F5C-6509E222F280

####### Literature record.

Cited from Morocco (Chandler 1994), based on 1 male in MNHN, which was from maison forestière de Khenolap-el-Ouaer, 1580 m, VII/1975, coll. J. Beaucournu.

####### New record.

Rif: Forêt-Jbel Lekraa, 1♂, 12/VI/2013, coll. Ebejer.

###### ** Mycetophila spectabilis

Taxon classificationAnimaliaDipteraMycetophilidae

Winnertz, 1864

E0B89CAD-1F58-5772-8112-01C7B7654416

####### New records.

Rif: Forêt-Jbel Lekraa, 1♂, 12/VI/2013, coll. Ebejer; Forêt-Aïn Boughaba, 1♂, 24/V/2013; Oued à 15 km de Fifi, 1♂, 06/V/2014; Aïn El Malâab, 1♂, 17/VI/2014.

###### * Mycetophila strigatoides

Taxon classificationAnimaliaDipteraMycetophilidae

(Landrock, 1927)

782F169D-5615-53DD-888E-BF67C2404F1B

####### Literature records.

Cited from North Africa (Tunisia) (Chandler 1994).

####### New records.

Rif: Forêt-Taghzoute, 1♂, 25/IV/2015, coll. Ebejer; Oued Aârate, 1♂, 26/III/2014.

###### ** Mycetophila unicolor

Taxon classificationAnimaliaDipteraMycetophilidae

Stannius, 1831

3C246349-3967-5369-8148-33A9D01FCAC5

####### New record.

Rif: Oued Kelâa, 1♂, 13/II/2013.

###### ** Mycetophila vittipes

Taxon classificationAnimaliaDipteraMycetophilidae

Zetterstedt, 1852

2E0F694C-D89D-5E82-BD42-31035A382376

####### New records.

Rif: Forêt-Jbel Lekraa, 2♂♂, 12/VI/2013, coll. Ebejer; Maison forestière, 1♂, 1♀, 18/V/2014, Daya Amsemlil, 7♂♂, 25/IV/2016.

##### Genus *Phronia* Winnertz, 1864

###### Phronia biarcuata

Taxon classificationAnimaliaDipteraMycetophilidae

(Becker, 1908)

F09F7896-B29B-5AAF-83D4-53F609C34FDB

####### Literature records.

Recorded from Morocco (Chandler 1994; Chandler and Ribeiro 1995, Chandler et al. 2005), based on specimens in MNHN, which were from Chefchaouen, VII/1975, coll. J. Beaucournu.

####### New records.

Rif: Daya Tazia, 1♂, 25/IV/2014; Aïn El Ma Bared, 1♀, 06/V/2014; Aïn El Malâab, 1♀, 17/V/2014; Aïn Takhninjoute, 1♂, 17/V/2014, 1♀, 21/IV/2015; Maison forestière, 1♂, 17/VI/2014; Grotte d’Hercule, 1♂, 29/III/2015; Daya Amsemlil, 4♂♂, 3♀♀, 23/IV/2016.

###### ** Phronia cinerascens

Taxon classificationAnimaliaDipteraMycetophilidae

Winnertz, 1864

A958C746-26C2-5E16-9C4D-C2B2DEE4C61C

####### New record.

Rif: Daya Amsemlil, 1♂, 28/II/2015.

###### ** Phronia nitidiventris

Taxon classificationAnimaliaDipteraMycetophilidae

(van der Wulp, 1858)

0E6907CE-DCBF-5686-8EB9-338AF5A0310B

####### New records.

Rif: Daya Afersiw, 1♂, 11/VI/2013; Oued Aârate, 1♂, 26/III/2014.

###### * Phronia tenuis

Taxon classificationAnimaliaDipteraMycetophilidae

Winnertz, 1864

37BD3A75-8ABA-52B6-823E-29CB41A8FAFE

####### Literature records.

Recorded from North Africa (Tunisia and Algeria) (Chandler 1994).

####### New records.

Rif: Oued Kelâa, 1♂, 1♀, 13/II/2013; Oued Aârate, 1♂, 3♀♀, 26/III/2014, 1♂, 07/V/2015; Oued Majjou (Majjou Village), 1♂, 10/V/2014; Aïn Takhninjoute, 2♂♂, 17/V/2014; Grotte d’Hercule, 1♂, 29/III/2015.

###### ** Phronia tyrrhenica

Taxon classificationAnimaliaDipteraMycetophilidae

Edwards, 1928

800BD493-0E21-5819-8F3C-4EE95B668C55

####### New records.

Rif: Forêt-Jbel Lekraa, 1♂, 12/VI/2013, coll. Ebejer; Maison forestière, 1♂, 18/V/2014; Daya Amsemlil, 2♂♂, 28/II/2015; Aïn Takhninjoute, 6♂♂, 5♀♀, 21/IV/2015; Daya Amsemlil, 3♂♂, 1♀, 23/IV/2016.

###### ** Phronia willistoni

Taxon classificationAnimaliaDipteraMycetophilidae

Dziedzicki, 1889

5B3CA63C-700C-560C-B18D-FCE82106AE04

####### New records.

Rif: Forêt-Jbel Lekraa, 1♂, 12/VI/2013, coll. Ebejer; Oued Aârate, 1♂, 26/III/2014; Maison forestière, 1♂, 1♀, 18/V/2014; Daya Amsemlil, 3♂♂, 3♀♀, 28/II/2015, 1♂, 1♀, 23/IV/2016; Cascade Chrafate, 1♂, 28/IV/2015; Bab El Karne, 14♂♂, 1♀, 25/XII/2015.

##### Genus *Sceptonia* Winnertz, 1864

###### ** Sceptonia intestata

Taxon classificationAnimaliaDipteraMycetophilidae

Plassmann & Schacht, 1990

7653A243-8805-5250-BE0D-B48078ADDE70

####### New records.

Rif: Maison forestière, 1♂, 17/VI/2014; Aïn El Malâab, 1♂, 17/V/2014.

###### ** Sceptonia membranacea

Taxon classificationAnimaliaDipteraMycetophilidae

Edwards, 1925

C1C83D10-8F02-5461-AB8A-5ECF1C80FB7F

####### New records.

Rif: Oued à 15 km de Fifi, 2♂♂, 06/V/2014; Oued Aârate, 1♂, 07/V/2015.

##### Genus *Trichonta* Winnertz, 1864

###### ** Trichonta foeda

Taxon classificationAnimaliaDipteraMycetophilidae

Loew, 1869

4F20244C-64F3-517F-A78D-327A5BB5DB19

####### New records.

Rif: Aïn Takhninjoute, 1♂, 21/IV/2015; Bab El Karne, 3♂♂, 1♀, 25/XII/2015; Daya Amsemlil, 1♂, 23/IV/2016; Oued Tkarâa, 1♂, 16/V/2016.

###### ** Trichonta icenica

Taxon classificationAnimaliaDipteraMycetophilidae

Edwards, 1925

EBE7AA01-2872-5A6D-B1B1-2A4D15C9DDC7

####### New record.

Beni Snassen: Grotte du chameau, 1♂, 24/XI/2015.

###### * Trichonta vitta

Taxon classificationAnimaliaDipteraMycetophilidae

(Meigen, 1830)

E07EA674-605B-529C-A200-B4F032758356

####### Literature records.

Recorded from North Africa (Algeria) (Hackman et al. 1988; Chandler 1994).

####### New records.

Rif: Forêt-Jbel Lekraa, 1♀, 12/VI/2013, coll. Ebejer; Bab El Karne, 1♂, 25/XII/2015.

###### ** Trichonta vulcani

Taxon classificationAnimaliaDipteraMycetophilidae

Dziedzicki, 1889

DC5CA663-8764-5A1D-91C9-C2FBDEB9E995

####### New record.

Beni Snassen: Grotte du Chameau, 1♂, 24/XI/2015.

##### Genus *Zygomyia* Winnertz, 1864

###### ** Zygomyia humeralis

Taxon classificationAnimaliaDipteraMycetophilidae

(Wiedemann, 1817)

F7EAF2C6-8C7A-5544-BC3D-7C8E6BFECB68

####### New record.

Rif: Maison forestière, 1♂, 17/VI/2014.

###### ** Zygomyia valida

Taxon classificationAnimaliaDipteraMycetophilidae

Winnertz, 1864

60E4D9EF-0FB5-52AE-A24C-EF2A944A3643

####### New records.

Rif: Aïn Ras El Ma, 1♂, 1♀, 3/V/2013; Oued Aârate, 1♂, 26/III/2014.

#### Subfamily Leiinae

##### Genus *Docosia* Winnertz, 1864

###### ** Docosia gilvipes

Taxon classificationAnimaliaDipteraMycetophilidae

(Walker, 1856)

F2CF8184-5183-54AB-A8E9-3A38F80E455D

####### New records.

Rif: Forêt-Jbel Lekraa, 1♂, 12/VI/2013, coll. Ebejer; Maison forestière, 1♂, 07/VI-17/X/2014, Malaise trap; Maison forestière, 1♂, 17/VI/2014; Aïn Takhninjoute, 1♂, 21/IV/2015; Daya Amsemlil, 1♀, 23/IV/2016.

##### Genus *Leia* Meigen, 1818

###### * Leia arsona

Taxon classificationAnimaliaDipteraMycetophilidae

Hutson, 1978

B28A9E5E-E1FC-575F-8AED-E5052A537672

####### Literature records.

Cited from North Africa (Tunisia) (Chandler 1994; Chandler and Gatt 2000).

####### New records.

Rif: Oued Majjou (Majjou Village), 1♂, 09/IV/2013; Oued Sidi Yahya Aârab, 1♀, 25/IV/2015. Middle Atlas: Aïn Walili, 1♂, 18/II/2016.

###### * Leia beckeri

Taxon classificationAnimaliaDipteraMycetophilidae

Landrock, 1940

F066E216-0845-5DD6-A9E7-064F543379FC

####### Literature records.

Cited from North Africa (Algeria) (Hackman et al. 1988; Chandler 2009).

####### New record.

Rif: Aïn Ras El Ma, 1♀, 27/III/2013.

###### Leia bimaculata

Taxon classificationAnimaliaDipteraMycetophilidae

(Meigen, 1804)

F6851B28-0874-553B-8615-92E7DCB4FB52

####### Literature records.

Cited from Morocco (Chandler et al. 2005), based on 1♂ and 1♀ from Forêt-Ifrane, 12/V/1961, coll. P.N. Lawrence (BMNH).

####### New records.

Rif: Daya Tazia, 1♂, 25/IV/2014; Maison forestière, 1♂, 07/VI-17/X/2014, Malaise trap; Aïn El Ma Bared, 1♂, 25/XII/2015. Middle Atlas: Forêt-3.5 km S. Azrou, 1♀, 8/V/2012, coll. Ebejer.

##### Genus *Novakia* Strobl, 1893

###### * Novakia scatopsiformis

Taxon classificationAnimaliaDipteraMycetophilidae

Strobl, 1893

67A231D4-19CA-5673-B82D-DD5178CA2ED7

####### Literature records.

Cited from North Africa (Tunisia) (Hackman et al. 1988; Chandler 1994).

####### New record.

Rif: Maison forestière, 10♂♂, 1♀, 07/VI-17/VI/2014, 1♂, 17/XI/2015, Malaise trap.

###### ** Novakia simillima

Taxon classificationAnimaliaDipteraMycetophilidae

Strobl, 1910

2C815B22-10FA-52CE-A212-C9F84DECD7EB

####### New records.

Rif: Oued Aârate, 1♂, 26/III/2014; Maison forestière, 11♂♂, 07/VI-17/VI/2014, 9♂♂, 17/XI/2015, Malaise trap.

#### Subfamily Gnoristinae

##### Genus *Boletina* Staeger, 1840

###### ** Boletina gripha

Taxon classificationAnimaliaDipteraMycetophilidae

Dziedzicki, 1885

3B7D7551-564E-55C9-80EF-CB07A9A00B94

####### New records.

Rif: Daya Fifi, 3♂♂, 23/XI/2012; Oued Aârate, 1♂, 26/III/2014; Daya Fifi, 1♂, 26/III/2014; Aïn Sidi Brahim Ben Arrif, 1♂, 25/IV/2014; Daya Amsemlil, 2♂♂, 28/II/2015; Grotte d’Hercule, 1♂, 29/III/2015, Cascade Chrafate, 2♂♂, 28/IV/2015; Oued Sidi Yahya Aârab, 1♂, 25/IV/2015; Aïn El Ma Bared, 2♂♂, 25/XII/2015; Bab El Karne, 2♀♀, 25/XII/2015. Middle Atlas: Forêt-3.5 km S. Azrou, 2♂♂, 8/V/2012, coll. Ebejer.

##### Genus *Coelosia* Winnertz, 1864

###### ** Coelosia fusca

Taxon classificationAnimaliaDipteraMycetophilidae

Bezzi, 1892

835D9D83-7E9F-5A28-9570-BFD52D923F7B

####### New records.

Rif: Daya Fifi, 1♀, 23/XI/2012; Oued Kelâa, 1♂, 13/II/2013; Daya Fifi, 1♂, 26/III/2014; Oued Amsemlil, 2♂♂, 2♀♀, 28/II/2015, 1♂, 31/I/2017; Aïn Takhninjoute, 1♂, 21/IV/2015; Cascade Chrafate, 2♂♂, 28/IV/2015; Bab El Karne, 1♀, 25/XII/2015; Daya Amsemlil, 1♀, 23/IV/2016; Daya avant Taïda, 2♂♂, 20/IV/2018.

##### Genus *Synapha* Meigen, 1818

###### ** Synapha fasciata

Taxon classificationAnimaliaDipteraMycetophilidae

Meigen, 1818

2540B3E3-77AF-5C46-879B-F2D17A1ECDAC

####### New records.

Rif: Aïn Sidi Brahim Ben Arrif, 1♂, 25/IV/2014; Daya Tazia, 1♂, 25/IV/2014; Forêt-Adrou, 1♀, 25/IV/2015, coll. Ebejer; Daya Amsemlil, 1♂, 23/IV/2016; Daya avant Taïda, 1♂, 20/IV/2018.

###### ** Synapha vitripennis

Taxon classificationAnimaliaDipteraMycetophilidae

(Meigen, 1818)

ABF4507A-6FAA-5649-8E87-0C0938C6E971

####### New record.

Rif: Daya Amsemlil, 1♂, 23/IV/2016.

##### Genus *Tetragoneura* Winnertz, 1846

###### ** Tetragoneura ambigua

Taxon classificationAnimaliaDipteraMycetophilidae

Grzegorzek, 1885

D8C5E489-FE71-50B3-98FE-5A165AD6A9C7

####### New records.

Rif: Forêt-Aïn Boughaba, 1♂, 24/V/2013. Beni Snassen: Oued Tafoughalt, 1♂, 25/XII/2014.

#### Subfamily Mycomyinae

##### Genus *Mycomya* Rondani, 1856

###### ** Mycomya flavicollis

Taxon classificationAnimaliaDipteraMycetophilidae

(Zetterstedt, 1852)

61F22551-1785-5E2E-8DE8-AFBDCED351A1

####### New records.

Rif: Aïn El Malâab, 1♂, 17/V/2014; Maison forestière, 40♂♂, 2♀♀, 07/VI-17/VI/2014, Malaise trap.

###### ** Mycomya pygmalion

Taxon classificationAnimaliaDipteraMycetophilidae

Väisänen, 1984

D7D27779-70AE-5488-92C5-4B411F105EFF

####### New records.

Rif: Oued Amsemlil, 6♂♂, 1♀, 28/II/2015; Aïn Sidi Brahim Ben Arrif, 2♂♂, 25/IV/2014.

###### ** Mycomya tumida

Taxon classificationAnimaliaDipteraMycetophilidae

(Winnertz, 1864)

5D779ADE-3E1E-5045-93B6-E61A8B542137

####### New record.

Rif: Daya Fifi, 2♂♂, 26/III/2014.

#### Subfamily Sciophilinae

##### Genus *Azana* Walker, 1856

###### * Azana anomala

Taxon classificationAnimaliaDipteraMycetophilidae

Staeger, 1840

D835E918-36D6-530D-AE62-556C1EF2CA8C

####### Literature records.

Cited from North Africa (Algeria) (Hackman et al. 1988).

####### New records.

Rif: Oued Majjou (Majjou Village), 1♂, 09/IV/2013; Maison forestière, 46♂♂, 1♀, 17/VI/2014.

##### Genus *Sciophila* Meigen, 1818

###### Sciophila iberolutea

Taxon classificationAnimaliaDipteraMycetophilidae

Chandler & Blasco-Zumeta, 2001

2FC3BCCE-9303-5674-AD56-A2A88D8EFB3B

####### Literature records.

Cited from Morocco: Rabat, Oued y Kern, V/1973, coll. H. Choumara, MNHN: (Chandler and Gatt 2000; Chandler and Blasco-Zumeta 2001, Bechev and Koç 2006).

####### New records.

Rif: Maison forestière, 1♂, 4/VII/2013, 4♂♂, 2♀♀, 07/VI-17/X/2014, Malaise trap, 1♂, 1♀, 17/VI/2014; Daya Jbel Zemzem, 1♂, 02/III/2015; Oued Sidi Yahya Aârab, 1♂, 25/IV/2015; Bab El Karne, 1♂, 25/XII/2015; Marabout El Khaloua, 1♂, 03/VI/2018.

## Discussion

The majority of the species recorded here are widespread in the Mediterranean region and also more widely in Europe and the Palaearctic Region. In addition to these, 10 species considered to be new to science were also found, and these will be treated elsewhere. These new species belong to the genera *Rymosia*, *Docosia*, *Leia*, *Megophthalmidia*, *Ectrepesthoneura*, and *Mycomya*; *Docosia* and *Megophthalmidia* in particular are diverse in the Mediterranean region, and the discovery of additional species in North Africa is not surprising. A female of *Allocotocera* resembling the Greek *A. scheria* Chandler, Bechev & Caspers, 2005 was also found, but males will be needed to establish if it is conspecific with *A. scheria*.

Knowledge of the North African Mycetophilidae is otherwise poor, with a relatively small number of species recorded from Algeria and Tunisia. Seven species were recorded from Algerian caves by Burghele-Balacesco (1967), while the citation of others (Chandler 1994; Chandler and Ribeiro 1995; Chandler and Gatt 2000; Chandler et al. 2005) was based on specimens in the collections of MNHN and BMNH. Altogether, only 21 species have been recorded from Algeria and 13 species from Tunisia, with a combined total of 31 species, of which 19 are in common with the present Moroccan list. The additional 12 species are all widespread in Europe, and can also be expected to occur in Morocco. The Atlantic Islands fauna was dealt with by Chandler and Ribeiro (1995), with four species added by Chandler and Báez (2002); of the 68 species recorded, 23 are in common with Morocco, while 27 are apparently endemic to the islands. Of the 39 species found in the Canary Islands, 16 are in common with Morocco and 13 are island endemics.

The Mycetophilidae of the Iberian Peninsula are much better known, with published records of 247 species, but this is a diverse region for which a direct comparison is not practical, and compared to other European countries, a much greater total can be expected with more recording. Not surprisingly, 56 of the species recorded here from Morocco are in common with the Iberian Peninsula. Otherwise, the study of the Mediterranean fauna has been piecemeal, but there have been detailed accounts of the fauna of Israel (Chandler 1994: 64 species, 28 also in Morocco), Malta (Chandler and Gatt 2000: 21 species, 11 in Morocco), Greece and Cyprus (Chandler et al. 2005: 151 species, 46 in Morocco; for Cyprus 54 species, 25 in Morocco) and Sardinia (Chandler 2009: 102 species, 44 in Morocco). Thus, more than 40% of the species for each studied country or region are in common with Morocco, except for the larger land areas of mainland Greece and the Iberian Peninsula where more central European species are expected to be present.

Moroccan species whose distribution is entirely or principally Mediterranean are the following, with the distribution outside Morocco stated in each case:

*Anatella concava* (Corsica, mainland Spain)

*Cordyla styliforceps* (Canary Islands, widespread in southern Europe, most northerly Switzerland and Ukraine (Crimea))

*Exechiopsis coremura* (Algeria, Corsica, Iberian Peninsula)

*Leia beckeri* (Canary Islands, Algeria, mainland Spain, Sardinia)

*Rymosia beaucournui* (Iberian peninsula, Sardinia, Greece including Lesbos and Crete, southern France and Switzerland)

*R. pseudocretensis* (Iberian Peninsula, southern France, mainland Italy, Crete)

*Phronia tyrrhenica* (mainland Spain, Corsica, Greece, Cyprus, southern France and Switzerland)

*Sceptonia intestata* (mainland Spain, Greece, Cyprus)

*Novakia simillima* (mainland Spain, most northerly Austria)

*Mycomya pygmalion* (Iberian Peninsula, Greece including Crete, Cyprus, Lebanon, Israel)

*Sciophila iberolutea* (mainland Spain, Malta).

## Supplementary Material

XML Treatment for Allodiopsis rustica

XML Treatment for Anatella concava

XML Treatment for Brevicornu intermedium

XML Treatment for Brevicornu griseicolle

XML Treatment for Brevicornu sericoma

XML Treatment for Brevicornu verralli

XML Treatment for Cordyla crassicornis

XML Treatment for Cordyla insons

XML Treatment for Cordyla murina

XML Treatment for Cordyla styliforceps

XML Treatment for Exechia bicincta

XML Treatment for Exechia dorsalis

XML Treatment for Exechia fulva

XML Treatment for Exechia fusca

XML Treatment for Exechiopsis coremura

XML Treatment for Pseudexechia tuomikoskii

XML Treatment for Rymosia affinis

XML Treatment for Rymosia beaucournui

XML Treatment for Rymosia pseudocretensis

XML Treatment for Stigmatomeria crassicornis

XML Treatment for Tarnania dziedzickii

XML Treatment for Mycetophila alea

XML Treatment for Mycetophila britannica

XML Treatment for Mycetophila deflexa

XML Treatment for Mycetophila edwardsi

XML Treatment for Mycetophila formosa

XML Treatment for Mycetophila marginata

XML Treatment for Mycetophila perpallida

XML Treatment for Mycetophila pictula

XML Treatment for Mycetophila sordida

XML Treatment for Mycetophila spectabilis

XML Treatment for Mycetophila strigatoides

XML Treatment for Mycetophila unicolor

XML Treatment for Mycetophila vittipes

XML Treatment for Phronia biarcuata

XML Treatment for Phronia cinerascens

XML Treatment for Phronia nitidiventris

XML Treatment for Phronia tenuis

XML Treatment for Phronia tyrrhenica

XML Treatment for Phronia willistoni

XML Treatment for Sceptonia intestata

XML Treatment for Sceptonia membranacea

XML Treatment for Trichonta foeda

XML Treatment for Trichonta icenica

XML Treatment for Trichonta vitta

XML Treatment for Trichonta vulcani

XML Treatment for Zygomyia humeralis

XML Treatment for Zygomyia valida

XML Treatment for Docosia gilvipes

XML Treatment for Leia arsona

XML Treatment for Leia beckeri

XML Treatment for Leia bimaculata

XML Treatment for Novakia scatopsiformis

XML Treatment for Novakia simillima

XML Treatment for Boletina gripha

XML Treatment for Coelosia fusca

XML Treatment for Synapha fasciata

XML Treatment for Synapha vitripennis

XML Treatment for Tetragoneura ambigua

XML Treatment for Mycomya flavicollis

XML Treatment for Mycomya pygmalion

XML Treatment for Mycomya tumida

XML Treatment for Azana anomala

XML Treatment for Sciophila iberolutea
